# Responses of Pigs of Different Genotypes to a Variation in the Dietary Indispensable Amino Acid Content in Terms of Their Growth, and Carcass and Meat Quality Traits

**DOI:** 10.3390/ani9080508

**Published:** 2019-07-31

**Authors:** Stefano Schiavon, Mirco Dalla Bona, Giuseppe Carcò, Enrico Sturaro, Luigi Gallo

**Affiliations:** Department of Agronomy, Food, Natural Resources, Animals and Environment (DAFNAE), University of Padova, Viale dell’Università 16, 35020 Legnaro (PD), Italy

**Keywords:** pigs, indispensable amino acids, growth performances, carcass quality, meat quality

## Abstract

**Simple Summary:**

The aim of the experiment was to study the response of pigs of different genotypes to a variation in their dietary amino acid allowance. Ninety-six crossbred barrows of two lean paternal genetic lines (Hypor Maxter and PIC 337) were housed in eight pens from body weight 60 to 145 kg and fed quasi ad libitum on feeds with either high or low ileal digestible indispensable amino acid contents. The high and low amino acid feeds were formulated to have the same standardized ileal digestible (SID) lysine, methionine, tryptophan and threonine contents per unit of crude protein, but provided 9.4 to 8.0 (assumed to be non-limiting) or 8.5 to 6.5 (close to National Research Council recommendations) g/kg of SID lysine, respectively. The low amino acid feed reduced the estimated N excretion compared with the high, without affecting growth, carcass yield, carcass anatomical composition and meat quality traits. Genetic line had trivial effects and the amino acid level × genotype interaction was never significant. We concluded that the two pig genotypes did not differ sufficiently in growth potential and leanness to respond differently.

**Abstract:**

We studied the response of pigs from two crossbred genetic lines (GL) in the 60 to 145 kg body weight interval to a variation in the indispensable amino acid (AA) content of their feed. Ninety-six barrows of two paternal GLs (Hypor Maxter and PIC 337) were housed in eight pens and fed *quasi ad libitum* on feeds differing in their standardized ileal digestible (SID) indispensable AA contents. Pigs in four pens received feeds containing 9.4 to 8.0 g/kg of SID Lys (HAA), considered non-limiting, while the others received feeds containing 8.5 to 6.5 g/kg of SID Lys (LAA). The two feeds had identical indispensable lysine, methionine, tryptophan and threonine contents per unit of crude protein (CP). Feed intake, growth, carcass weight, and the weights of the lean and fat cuts were recorded, and samples of *longissimus lumborum* were analyzed. Data were analyzed using a two-way factorial mixed model. The LAA feed lowered the estimated N excretion (*p* < 0.001) compared with HAA, without affecting growth, carcass or meat quality traits. Genetic line had trivial effects and the AA level × genotype interaction was never significant. The two pig genotypes did not differ sufficiently in growth potential and leanness to respond differently.

## 1. Introduction

Pigs of different genotypes may differ in their potential for lean growth, which affects their protein and amino acid (AA) requirements [1]. If pigs of different genotypes are given the same feed, their AA supply may be either greater or lower than their requirements at different points in their growing curve. When the AA supply is greater than required, nutrients are wasted and the resulting increase in N excretion has a negative impact on the environment [2]. Conversely, if the AA supply is less than required, growth, particularly of the lean body components will be impaired [3]. Pigs selected for lean growth are expected to have greater protein requirements and to utilize AA more efficiently for protein retention and growth [4]. He et al. (2016) [5] showed that low-protein diets positively affect ileal amino acid digestibility and the gene expression of digestive enzymes in growing and finishing pigs. When nutrient supplies are limiting, their partition among body constituents and functions tends to be similar regardless of genotype [6,7]. However, when pigs of different genetic backgrounds are fed non-limiting diets, the differences between genotypes may be more apparent, a pattern that would be evidenced by a feed × genotype interaction.

Despite being of great interest to the pig industry, only a few studies have so far investigated the response of pigs of different genotypes to a reduction in dietary AA. Fabian et al. (2002) [8] found few interactions as a result of restricting the AA content in the diets of different Duroc pig lines, either selected for lean growth efficiency or not. They found some feed × genotype interactions in feed efficiency and lysine utilization for weight gain in the later stage of growth, i.e., at a body weight (BW) in the range 80–108 kg. This would appear to reflect some divergences in the characteristics of the growth curves and, in turn, of nutrient requirements at the heaviest body weights.

The aim of the current study, therefore, was to evaluate growth performance, and carcass and meat quality traits of fast-growing pigs from two genetic lines fed diets containing either high or reduced amounts of crude protein (CP) and indispensable AA until they reached 145 kg BW and to look for possible feed × genotype interactions.

## 2. Materials and Methods

All experimental procedures were reviewed and approved by the Ethical Committee for the Care and Use of Experimental Animals of the University of Padova (CEASA, #147683). 

### 2.1. Animals, Feeding and Experimental Design

The trial involved 96 crossbred barrows from the same maternal line (PIC-Camborough) but from two different commercial paternal lines selected for improved growth rate, feed efficiency and yield of lean cuts (Hypor Maxter and PIC 337). The pigs were provided by a feed industry. They came from the same farm, were born in the same week and were fed the same feed until they were moved to the experimental station at an average body weight of 30 ± 2 kg.

On arrival, the pigs were housed in eight pens (12 pigs per pen, balanced for BW and genetic line) measuring 5.8 × 3.8 m and with fully slatted floors. Each pen was equipped with a single-space electronic feeder (Compident Pig–MLP, Schauer Agrotronic, Prambachkirchen, Austria) programmed to supply the pigs with the planned daily amount of feed and to record the amount of feed eaten by individual pigs, in accordance with Schiavon et al. (2018) [9]. During the first 28 d of trial (acclimation) all pigs were given the same feed (Table 1), which provided 13.9 MJ/kg metabolizable energy (ME), 10.2 MJ/kg net energy (NE), 164 g/kg CP and 11.0 g/kg standardized ileal digestible lysine (SID Lys) (Table 2). From the 29th d on feed, the pigs in 4 pens were given the high AA/high CP feeds (HAA), which provided 13.9 MJ/kg ME, 10.1 MJ/kg NE, 163 g/kg CP and 9.4 g/kg SID lysine during the growing period (29 to 70 d on feed, 60 to 104 kg BW), and 158 g/kg CP and 8.0 g/kg SID lysine during the finishing period (71 to 118 d on feed, 104 to 145 kg BW). The HAA feeds provided amounts of the four main indispensable AAs in excess of those recommended by the NRC (2012) [10].

The pigs in the remaining four pens were given feeds with lower AA contents (LAA), which were formulated from the corresponding HAA feeds by replacing soybean meal with corn grain and adding crystalline AA in order to match the contents and the ratios of the main indispensable AAs (lysine, methionine, threonine, tryptophan) per CP unit in the two feeds. The LAA feeds provided the same amount of ME and NE as the HAA feeds (13.9 and 10.1 MJ/kg, respectively), but nearly 10% less CP and SID indispensable AA during the growing stage (146 and 8.5 g/kg, respectively) and nearly 20% less CP and SID indispensable AA during the finishing stage (126 and 6.5 g/kg, respectively). The indispensable AA contents of the LAA feeds were similar to those recommended by the NRC (2012) [10]. When expressed per unit of CP, the proportions of the various indispensable amino acids were almost identical in the HAA and LAA feeds. The pigs of two genotypes passed from the early to the late period feeds contemporaneously.

All pigs received daily feed amounts established on a weekly basis according to a feeding curve described in Schiavon et al. [9]. This feeding curve has been developed for medium- heavy-weight pigs (145 kg body weight) providing an average feed allowance equal to 95% of the ad libitum feed intake. This plan was devised to optimize feed efficiency by preventing excessive consumption by the greedier pigs [11,12]. Water was freely available from a nipple drinker placed in each pen.

The pigs were weighed every week using electronic scales. Backfat (BF) thickness was measured every three weeks using an A-mode ultrasound device (Renco Lean-Meter series 12, Renco Corporation, Minneapolis, MN, USA) applied above the last rib approximately 5.5–8.0 cm from the midline, the greater the BW, the greater the distance [13]. During the trial, four pigs died or were discarded because of injuries (three on the LAA and one on the HAA diet). The corresponding data were removed, so that the final dataset was drawn from 92 pigs.

### 2.2. Slaughter and Data Collection

When the pigs reached the average target BW of 145 kg, they were fasted for 24 h then taken to a commercial abattoir and slaughtered in accordance with commercial practices. The pigs were stunned with a high concentration of CO_2_, then jugulated and exsanguinated. The carcasses were scalded, de-haired, eviscerated and split along the midline. Hot weight was recorded online and the lean percentage [14,15] was measured by image analysis of the left side (CSB-Image-Meter^®^, CSB-System AG, Geilenkirchen, Germany). Hot carcasses were processed according to the usual procedures and the loin without ribs, the shoulder, thigh, backfat and belly were weighed. A section of *longissimus lumborum* (LL), including the last two lumbar vertebrae, was collected from the left loin of each carcass, placed in individual plastic bags, refrigerated for 24 h, then vacuum-packed at −20 °C pending analyses.

### 2.3. Feed Analysis

The feeds were produced from the same batches of ingredients. Ten samples of each feed were collected online during manufacture, pooled and sub-sampled into three independent aliquots of 300 g each. These sub-samples were analyzed for dry matter, N, ether extract and ash (methods 934.01, 976.05, 920.29 and 942.05, respectively [16]), and for neutral detergent fiber [17]. Starch content was determined after hydrolysis to glucose by liquid chromatography [18]. Dietary ME, SID AA and other nutrients were computed from the actual ingredient composition of the feeds and from the tabular values [10] of each ingredient.

### 2.4. Meat Analysis

Muscle pH was measured in triplicate on the LL sample 45 min and 24 h after slaughter using a Crison Basic 25 portable pH meter equipped with a Crison 5033 penetration probe (Crison, Barcelona, Spain). Frozen samples of LL were thawed in vacuum-packaged bags for 24 h at 4 °C, then removed from the packaging, blotted and weighed. Cooking loss was measured on a 2.5 cm-thick subsample of LL, which was weighed and sealed in a plastic bag, cooked in a water bath at 75 °C for 50 min to a core temperature of 70 °C and then cooled to room temperature, blotted dry, and weighed again. Cooking loss percentage was computed by dividing the difference between the pre- and post-cooked weights by the pre-cooked weight. Five cylindrical cores of 1 cm^3^ were collected from the same subsample and sheared perpendicularly with a Lloyd^®^ (Bognor Regis, UK) LS5 series Warner-Bratzler shearing device (shearing velocity 1 mm/s) using the NEXIGEN Plus 3 software. Another subsample of LL was ground and homogenized for 10 s at 4500 g (Grindomix GM200; Retsch, Haan, Düsseldorf, Germany) for chemical analyses. Moisture was determined by leaving overnight in an oven at 101–103 °C (method 950.46; AOAC 2012) [16]; crude protein (CP) was measured by multiplying the organic N content by 6.25 (method 976.05 [16]); fat was determined by extraction with petrol ether (method 991.36; [16]); and ash was determined by mineralization in a muffle furnace at 550 °C (method 920.153; [16]).

### 2.5. Data Editing

Average daily gain (ADG), daily feed intake and the gain to feed ratio (G:F) were calculated from the data. Nitrogen flow was estimated as described by Gallo et al. (2014) [3]. Briefly, empty BW (EBW) was estimated from BW using the equation provided by Kloareg et al. (2006) [12]. Body lipid mass (BL, kg) was estimated from BF and BW [12]. Fat-free EBW mass (FFEBW) was computed as EBW minus BL. Body protein mass (kg) was computed with an allometric equation from FFEBW, according to the NRC (2012) [10]. Protein retention (Pr) was computed from changes in body protein mass. Daily N excretion was determined as N intake-N retention (NR), where N intake was calculated from the feed intake and feed N content, and NR was estimated from body Pr (Pr/6.25).

### 2.6. Statistical Analysis

All data analyzed using the SAS MIXED procedure (SAS Institute Inc., Cary, NC) according to the following linear model:

*y_ijkl_* = μ + GL*_i_* + Feed*_j_* + pen(Feed)_*k*:*j*_ + (GL × Feed)*_ij_* + *e_ijkl_* (1)

where *y_ijkl_* is the observed trait, μ is the overall intercept of the model, GL*_i_* is the fixed effect of the *i*th genetic line (*i*: 1 = A, 2 = B), Feed_*j*_ is the fixed effect of the *j*th feed (*j*: 1 = HAA, 2 = LAA), pen(Feed)_*k*:*j*_ is the random effect of the *k*th pen (*k* = 1, …, 8) within feed, (GL × feed)*_ij_* is the effect of the interaction between genetic line and feed, and *e_ijkl_* is the random residual. Pen and the residuals were assumed to be independently and normally distributed with mean zero and variances of $\sigma_{1}^{2}$ and $\sigma_{e}^{2}$, respectively. In line with the experimental design, the effect of Feed was tested using pen (Feed) as the error line, whereas the effect of GL was tested on the residual (individual) as the error line, given that pigs of both GLs were housed in the same pen. 

## 3. Results

### 3.1. Growth Performance and Estimated Lipid and Protein Retention

The pigs reached an average BW of 145 kg after 118 d on feed, and their ADG from the start of the growing phase (around 60 kg BW) to the end of the trial averaged 0.95 kg/d (Table 3).

**Figure 1:**
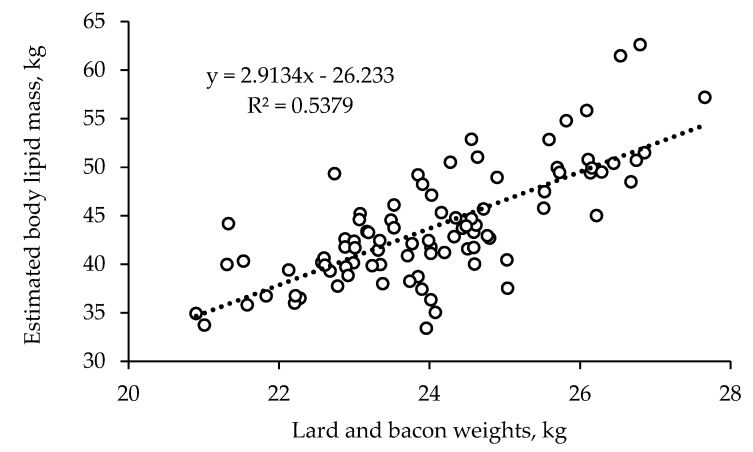
Correspondence between weights of the lard and bacon and body lipid mass estimated from in vivo measurement of body weight and backfat depth.

In the same interval, feed intake averaged nearly 2.7 kg/d and the resulting feed efficiency (gain:feed) averaged 356 g/kg, corresponding to a feed conversion ratio of about 2.80. In general, the dietary AA content did not affect growth performances. The pigs on the LAA diets had nearly identical final BW, growth rates and feed efficiency to the HAA pigs. However, pigs of the PIC 337 genetic line had a 3% better feed conversion rate (gain:feed) to pigs of the Hypor Maxter genetic line (*p* = 0.05), due almost entirely to their small, but significantly greater growth rate (3%, *p* = 0.022). No interaction between the AA content of the feed and genetic line was observed for growth performance. The final body lipid and protein masses averaged 44.1 and 21.7 kg, respectively, with no significant difference due to dietary AA content or genotype. There was a tendency toward a dietary AA content × genotype interaction (*p* = 0.09) due to the body lipid mass of the Hypor Maxter barrows on the HAA feed (34.9 kg) being slightly lower than the final lipid mass with all the other treatments (in the order of 36.8 kg). This arose from the slightly lower average lipid retention of the Hypor Maxter pigs on the HAA diet (300 g/d) compared with the other groups (334 g/d) (*p* = 0.06). The estimated protein retention averaged 182 g/d during growing and 102 g/d during finishing, uninfluenced by diet, genetic line or their interaction. The estimated N retention per kg of BW gain averaged 23.7 g/kg. There was a good correlation (R^2^ = 0.53) between individual body lipid mass, estimated from BW and BF depth measured in vivo, and the sum of the lard and bacon weights recorded at the slaughterhouse (Figure 1).

### 3.2. Nitrogen Flow

The reduction in the CP content of the diets resulted in a comparable reduction in N intake, which was on average 10% lower in the LAA than the HAA pigs during growing (*p* < 0.001) and 20% lower during finishing (*p* < 0.001) (Table 4). As the estimated N retention was nearly identical in the pigs on the different feeds, the reduction in the dietary CP content from 60 kg BW onwards resulted in a nearly 24% reduction in the estimated N excretion over the same period. The pigs of the two genotypes responded in the same way to the lowering of the dietary CP content, and genotype did not affect the estimated N retention and excretion.

### 3.3. Carcass and Meat Quality

Carcass weight (Table 5) averaged 115 kg and dressing yield was close to 79%. In general, the differences in the AA contents of the feeds did not affect the carcass traits of the pigs. The LAA pigs yielded carcasses of the same weight as the HAA pigs and with a comparable lean meat percentage, but the loin yield tended to be nearly 2% lower (*p* = 0.06). Pigs of the different genotypes also exhibited similar carcass traits and yield of main cuts and responded alike to the lowering of the indispensable AA content of the feeds. There was no evidence that the feed × genotype interaction influenced the carcass traits. The physical and chemical characteristics of the LL were comparable in pigs of different genotypes on diets with different CP contents, and here, too, the interaction between feed and genotype was never significant (Table 6).

## 4. Discussion

### 4.1. Dietary Supply of Indispensable Amino Acids

In the current experiment, the pigs were fed controlled amounts of feed from 60 to 145 kg BW in order to improve feed efficiency and carcass uniformity at slaughter [19]. Growth performances were good, the daily gain averaged 0.95 kg/d and the gain:feed ratio was 0.357, in agreement with previous studies conducted under similar conditions [20]. In a study with pigs fed *ad libitum* and with lighter BW ranges (30–90 kg), Ball et al. (2013) [21] reported a growth rate of 0.94 kg/d and a gain:feed ratio of 0.406, while similar figures were reported by Morales et al. (2011) [22].

Protein retention, estimated from in vivo measurements of body weight and of backfat depth by ultrasound, averaged 182 g/d during growing and 102 g/d during finishing. The former value is greater than and the latter lower than those recommended by the NRC (2012) [10] for high-medium lean growth rates, which are 147, 141 and 122 g/d in the BW ranges of 50–75, 75–100 and 100–135 kg BW, respectively. Ruiz-Ascacibar et al. (2017) [23] reported mean protein deposition rates in barrows of 142, 153, 147, 141 g/d at BWs of 80, 100, 120 and 140 kg, respectively. The estimated N retention averaged 23.3 g/kg of body gain, slightly lower than the value (25 g/kg) calculated elsewhere [24] for pigs slaughtered at lighter weights (90–120 kg), and consistent with the value of 24 g/kg calculated by others for heavy pigs of about 160 kg body weight [25,26]. There was also good correspondence between the body lipid mass estimated from BW and BF depth measured by ultrasound and the weights of the lard and bacon, the main fat components of the carcasses. These figures suggest that the pigs in the current experiment had a high potential for lean growth at early BWs, particularly between 60 and 100 kg.

In the current experiment, the indispensable AA allowance, expressed per kg of feed, was greater (HAA) or similar (LAA) to that recommended by the NRC (2012) [10] for pigs with high-medium lean growth rates. The higher AA content was tested to avoid limiting conditions with respect to the indispensable AA daily allowance, given the pigs’ high potential for lean growth and the possibility of the feed intake being lower than that recommended by the NRC (2012) [10]. Based on the actual feed intakes, we calculated the average SID Lys intake as 24.7 g/d (growing) and 22.3 g/d (finishing) for the HAA pigs, and 22.3 g/d (growing) and 18.0 g/d (finishing) for the LAA pigs. According to the NRC (1998) [27], 0.123 g of true digestible lysine per g of protein accretion is required above maintenance. On the basis of this recommendation, we calculated the total Lys requirements for maintenance and protein accretion to be about 26 g/d during growing and 15 g/d during finishing. The HAA feed provided 27.4 g/d (growing) and 24.9 g/d (finishing) of total lysine, the LAA feed 24.7 and 20.0 g/d, respectively. In the current experiment, the indispensable AA allowance had little or no influence on growth performance, body protein (or N) retention, feed efficiency, carcass characteristics and meat quality parameters. As the highest dietary AA contents produced no benefits, we suggest that they were greater than requirement and that, therefore, the lowest dietary AA content can be used with pigs similar to those tested in the current experiment to reduce feed costs and lower N excretion into the environment. Given the nature of the estimated protein growth, a further reduction in indispensable AA contents may be possible, particularly in the 104–145 kg BW range.

### 4.2. Feed × Genotype Interaction

The current experiment did not aim to compare the two genetic lines, but rather to explore their possible interaction in response to a variation in the indispensable AA content of the feed. There are very few comparisons between pig genetic lines in the literature [28], but the potential for lean growth is known to largely differ among genotypes [6]. Emphasis on traits such as growth rate and subcutaneous fat affects the mass of metabolically active organs, feed intake, protein retention, activity of lipogenic enzymes, plasma concentrations of hormones and metabolites, and metabolism of adipose tissues [8]. The two genetic lines used in the current experiment were selected for the production of both fresh meat and high-quality cooked hams [29,30], which requires fast growing pigs slaughtered at about 135–145 kg BW to obtain untrimmed thighs of about 14–16 kg [20]. The Hypor Maxter pigs used in the current experiment were selected for fast growth, carcass leanness, and feed efficiency [31], while the PIC 337 were selected for fast growth, feed efficiency, lower P2 backfat depth, carcass yield and conformation [32]. There is little information in the scientific literature on these two genetic lines.

The two GLs used in the current study differed significantly in growth performances, although the difference was slight-close to 30 g/d, and arose under conditions of controlled feeding, likely reflecting a difference in feed efficiency, which was 2% better in the PIC 337 than in the Hypor Maxter pigs. Conversely, the carcass yield of the Hypor Maxter was greater than that of the PIC 337 pigs. The carcasses of the two genetic lines were very similar in the composition of the anatomical cuts and in their meat quality characteristics.

Kyriazakis (2011) [6] suggested that when the nutrient supplies are limiting, their partition among body constituents and functions tends to be similar among genotypes, so that high, non-limiting protein supplies are required to point up the differences among genotypes. Taylor et al. (2015) found that Hampshire piglets grew faster than Large White pigs when fed a non-lysine-limiting diet but grew at similar rates when fed a lysine-limiting diet, indicating that dietary lysine level rather than genotype determined their growth performance. Chiba et al. (2002) [33] observed some diet × genotype interactions, suggesting that fast-growing, lean pigs need to be provided with adequate indispensable AA concentrations. They also concluded that compensatory growth responses are influenced by pig genotype.

Ruiz-Ascacibar et al. (2017) [23] found a significant diet × sex interaction in a trial involving entire and castrated males and entire females fed ad libitum diets that differed in protein content. In particular, in the 100 to 140 kg BW interval entire males fed a low protein diet increased their feed intake by 20% compared with entire males fed the control diet. In contrast, castrated males and females reduced their feed intake when fed the low protein diet. This interaction may be due to the different shapes of the protein deposition curves, and the authors also attributed it to the extra energy required for castrated males and females to eliminate the excess protein. They also reported that the highest protein deposition rates were found with BWs heavier than those indicated by the NRC (2012), namely 168 (at 140 kg BW) for entire males, 153 (at 100 kg BW) for castrated males and 148 g/d (at 120 kg BW) for gilts. These results would suggest that different response to a given feed would be possible among entire males, castrated males and females of the same genotype. However, the pigs of two strains studied by De Greef (2002) [34] responded similarly to protein deficiency from 28 to 65 kg BW and re-alimentation from 66 to 105 kg BW. These authors suggested that the two strains of pigs did not differ sufficiently in leanness to respond differently, a finding similar to ours.

In fact, in the current experiment, the HAA diet was formulated to contain an amount of indispensable AA in excess of the NRC’s (2012) [10] recommendation, so that the potential for protein growth could have been expressed. The growth performances and carcass characteristics of the pigs of the two genetic lines were very similar whether the pigs were on the HAA or the LAA diet, and little or no interaction was found. However, unlike the previous experiments, the pigs of the current trial were fed controlled amount of feed, a feeding regime that may have limited their energy allowance and, in turn, limited their growth rate. Actual performance shows that when BW was over 104 kg the indispensable AA supply was probably in excess of requirements with both the HAA and the LAA diets, particularly when BW was in the range of 104–145 kg. This might have prevented a diet × genotype interaction emerging. However, it also suggests that the dietary indispensable AA content can probably be further reduced in the 104–145 BW interval.

## 5. Conclusions

Under the conditions of the current experiment, the variation in dietary indispensable AA content had almost no consequences for growth performance, carcass composition and meat quality, suggesting little or no interference in the chemical and anatomical growth of the pigs of the two genotypes. We conclude that the two pig genetic lines did not differ sufficiently in leanness to respond differently. It was also concluded that the indispensable AA contents of the LAA diets were adequate for the kind of growing pigs used in this experiment, as no benefits were achieved when their contents were increased with the HAA diets.

## Figures and Tables

**Table 1:** Ingredient composition of the experimental feeds (g/kg as fed).

Ingredients	Acclimation (0–28 d)	Growing (29–70 d)		Finishing (71–118 d)	
		High Amino Acid (HAA)	Low Amino Acid (LAA)	HAA	LAA
Corn grain	317.2	400.0	440.7	374.6	440.9
Wheat	280.0	280.0	280.0	350.0	350.0
Soybean Meal	135.0	120.0	75.0	110.0	25.0
Barley	120.0	-	-	-	-
Distillers’ dried grains	40.0	60.0	60.0	50.0	60.0
Wheat middling	40.0	60.0	60.0	50.0	50.0
Beef tallow	27.0	24.0	23.0	20.0	18.0
Sunflower	-	25.0	30.0	20.0	30.0
Calcium carbonate	13.0	12.0	12.0	13.0	13.0
Sodium chloride	5.0	5.0	5.0	5.0	5.0
Organic acids	5.0	4.0	4.0	-	-
Dicalcium phosphate	3.4	-	-	-	-
Vitamin and mineral premix ^1^	2.5	2.5	2.5	2.5	2.5
L-Lysine HCl	7.0	5.1	5.4	3.6	4.3
DL-Metionine	1.6	0.6	0.5	-	-
L-Threonine	2.4	1.4	1.4	1.0	1.0
L-Tryptophan	0.4	0.1	0.2	-	-
Choline HCl	0.5	0.3	0.3	0.3	0.3

^1^ Providing per kg of diet: 9000 UI of vitamin A, 2000 UI of vitamin D_3_, 1.5 mg of B_1_, 4 mg of vitamin B_2_, 3 mg vitamin B_6_, 20 mg of vitamin B_12_, 30 mg of vitamin E, 2.1 mg of vitamin K_3_, 22.5 mg of pantothenic acid, 25 mg of niacin, 0.3 mg of folic acid, 0.3 mg of biotin, 50 mg of Mn, 113 mg of Zn, 125 mg of Fe, 17.5 mg of Cu, 1.75 mg of J, 0.375 mg of Se.

**Table 2:** Chemical composition (g/kg as fed) and energy content of the experimental feeds.

Ingredient	Acclimation (0–28 d)	Growing (29–70 d)		Finishing (71–118 d)	
		High Amino Acid (HAA)	Low Amino Acid (LAA)	HAA	LAA
Analysed nutrient composition ^1^					
Dry Matter	898	891	891	889	888
Crude Protein (N × 6.25)	160	158	140	150	120
Starch	514	526	548	530	561
NDF	130	103	111	135	134
Ether Extract	59	55	52	44	42
Ash	44	39	36	40	37
Calculated nutrient composition ^2^					
Dry Matter	885	883	883	883	882
ME, MJ/kg	13.6	13.6	13.6	13.6	13.6
NE, MJ/kg	10.2	10.1	10.1	10.1	10.1
Crude Protein (CP)	164	163	146	158	126
Starch	439	442	467	457	503
Ca	6.6	5.4	5.3	5.8	5.6
P	4.4	4.0	3.9	4.0	3.8
Lysine	12.0	10.5	9.5	9.0	7.3
Methionine	4.0	3.1	2.8	2.5	2.1
Threonine	7.7	6.8	6.1	6.3	5.0
Tryptophan	2.3	2.0	1.8	1.9	1.4
SID Lysine, mg/g CP ^3^	67	58	58	51	52
SID Methionine, mg/g CP ^3^	23	17	17	14	14
SID Threonine, mg/g CP ^3^	41	36	36	34	33
SID Tryptophan, mg/g CP ^3^	12	10	10	10	10

^1^ Analytical results obtained by averaging data on 3 independent replications. ^2^ According to (NRC, 2012) [10]. ^3^ SID: Standardized ileal digestible.

**Table 3:** Growth performance of barrows of two genetic lines fed high (HP) or low (LP) indispensable amino acids feeds.

Traits	Feed (F)				Genetic Line (GL)				F × GL
	HAA	LAA	SEM	*p*	Hypor Maxter	PIC 337	SEM	*p*	*p*
Body weight, kg:									
0 d arrival	30.2	30.7	0.29	0.24	30.1	30.8	0.29	0.13	0.25
29 d (start of growing)	59.9	59.0	0.59	0.95	59.3	58.6	0.53	0.28	0.98
70 d (start of finishing)	104.0	103.7	0.77	0.76	103.6	104.5	0.72	0.65	0.46
118 d (end of trial)	145.6	144.5	1.04	0.48	144.3	145.9	0.98	0.19	0.63
Growth rate, kg/d:									
Growing	1.07	1.07	0.009	0.57	1.06	1.08	0.009	0.025	0.43
Finishing	0.87	0.85	0.015	0.50	0.85	0.87	0.013	0.08	0.16
Overall	0.96	0.95	0.008	0.35	0.94	0.97	0.008	0.022	0.95
Feed intake, kg/d:									
Growing	2.61	2.60	0.015	0.70	2.61	2.60	0.013	0.57	0.21
Finishing	2.77	2.75	0.027	0.67	2.76	2.76	0.021	0.89	0.25
Overall	2.69	2.68	0.017	0.60	2.69	2.68	0.014	0.86	0.15
Gain:feed:									
Growing	0.412	0.410	0.003	0.78	0.405	0.417	0.003	0.008	0.91
Finishing	0.313	0.310	0.003	0.43	0.307	0.316	0.003	0.039	0.43
Overall	0.358	0.355	0.003	0.52	0.351	0.362	0.003	0.005	0.19
Final body lipid mass, kg	43.9	44.3	1.25	0.63	43.7	44.5	1.09	0.60	0.09
Final body protein mass, kg	21.9	21.5	0.21	0.81	21.6	21.8	0.21	0.52	0.28
Lipid retention^1^, g/d									
Growing	300	304	32	0.78	302	302	30	0.97	0.66
Finishing	411	422	110	0.75	405	428	95	0.28	0.26
Overall	359	368	119	0.66	352	374	103	0.10	0.06
Protein retention ^1^, g/d									
Growing	183	181	10	0.62	179	184	10	0.12	0.33
Finishing	100	103	17	0.27	104	109	20	0.90	0.21
Overall	141	137	21	0.28	139	139	21	0.88	0.14
N retention^1^ g/kg BW gain	23.5	23.1	1.5	0.58	23.6	22.9	1.3	0.45	0.66

^1^ Estimated using BW and the ultrasound backfat thickness measured at the P2 point according to Kloareg et al. (2006) [13] and Gallo et al. (2014) [3].

**Table 4:** Estimated N flow of barrows of two genetic lines fed high (HP) or low (LP) indispensable amino acids feeds.

Traits	Feed (F)				Genetic Line (GL)				F × GL
	HAA	LAA	SEM	*p*	Hypor Maxter	PIC 337	SEM	*p*	*p*
Nitrogen intake ^1^, kg/pig:									
Growing step	2.84	2.54	0.014	<0.001	2.68	2.70	0.014	0.36	0.27
Finishing step	3.33	2.66	0.018	<0.001	2.99	3.00	0.018	0.70	0.44
Overall	6.17	5.20	0.032	<0.001	5.67	5.70	0.030	0.46	0.97
Estimated N retention, kg/pig ^2^:									
Growing step (42 d on feed)	1.26	1.26	0.015	0.98	1.25	1.28	0.015	0.18	0.16
Finishing step (47 d on feed)	1.00	0.95	0.029	0.20	0.97	0.99	0.024	0.48	0.95
Overall (89 d on feed)	2.27	2.21	0.030	0.21	2.22	2.27	0.030	0.25	0.94
Estimated N excretion, kg/pig ^3^									
Growing step	1.58	1.28	0.020	<0.001	1.44	1.43	0.020	0.70	0.09
Finishing step	2.32	1.71	0.042	<0.001	2.02	2.01	0.035	0.83	0.56
Overall	3.90	2.98	0.052	<0.001	3.45	3.44	0.047	0.75	0.20
N efficiency	0.368	0.417	0.003	< 0.001	0.390	0.395	0.003	0.12	0.57

^1^ Computed from feed intake and its N content. ^2^ Estimated using BW and the ultrasound backfat thickness measured at P2 level according to Kloareg et al. (2006) [13] and Gallo et al. (2014) [3]. ^3^ Computed as N intake-estimated N retention.

**Table 5:** Carcass traits of barrows of two genetic lines fed high (HP) or low (LP) indispensable amino acids feeds.

Traits	Feed (F)				Genetic Line (GL)				F × GL
	HAA	LAA	SEM	*p*	Hypor Maxter	PIC 337	SEM	*p*	*p*
Carcass:									
Weight, kg	115.0	114.9	0.65	0.92	114.7	115.2	0.65	0.60	0.99
Yield, %	79.0	79.5	0.01	0.16	79.6	79.0	0.002	0.08	0.15
Lean percentage ^1^, %	53.3	53.0	0.54	0.65	53.4	52.9	0.54	0.49	0.42
Untrimmed lean and fat cuts, kg:									
Loin	13.1	12.8	0.12	0.11	12.8	12.9	0.13	0.55	0.99
Ham	29.2	29.3	0.25	0.86	29.3	29.2	0.25	0.77	0.72
Total lean cuts	57.9	57.6	0.43	0.65	57.6	57.9	0.43	0.68	0.64
Total fat cuts	23.8	24.3	0.35	0.31	23.9	24.2	0.35	0.31	0.74
Yield of untrimmed lean and fat cuts, % of carcass:									
Loin	11.3	11.1	0.09	0.06	11.2	11.2	0.09	0.72	0.99
Ham	25.4	25.5	0.17	0.75	25.5	25.3	0.17	0.28	0.66
Total lean cuts	50.3	50.1	0.30	0.65	50.2	50.2	0.29	0.97	0.33
Total fat cuts	20.7	21.2	0.27	0.29	21.0	20.9	0.27	0.55	0.79

^1^ Assessed using CSB-Image-Meter (EU, 2014) [14,15].

**Table 6:** Longissimus lumborum (LL) physical and chemical characteristics in barrows of two genetic lines fed high (HAA) or low indispensable amino acid feeds.

Traits	Feed (F)				Genetic Line (GL)				F × GL
	HAA	LAA	SEM	*p*	Hypor Maxter	PIC 337	SEM	*p*	*p*
pH of LL ^1^:									
45 m	6.1	6.0	0.03	0.54	6.0	6.1	0.03	0.85	0.06
24 h	5.5	5.5	0.01	0.12	5.5	5.5	0.01	0.15	0.90
LL content of:									
Moisture, %	72.3	72.6	0.36	0.65	72.5	72.5	0.28	0.84	0.56
Protein, %	22.8	22.8	0.31	1.00	22.8	22.8	0.23	0.65	0.61
Intramuscular fat, %	3.5	3.2	0.16	0.33	3.5	3.3	0.15	0.39	0.88
Ash, %	1.17	1.17	0.02	1.00	1.16	1.17	0.01	0.26	0.23
LL cooking losses, %	34.0	35.2	0.53	0.17	34.6	34.6	0.44	0.80	0.83
LL shear force, kg	3.2	3.5	0.16	0.21	3.20	3.53	0.13	0.16	0.43

^1^ pH of LL collected 45 m and 24 h after slaughter.
